# A Gait Sub-Phase Switching-Based Active Training Control Strategy and Its Application in a Novel Rehabilitation Robot

**DOI:** 10.3390/bios15060356

**Published:** 2025-06-04

**Authors:** Junyu Wu, Ran Wang, Zhuoqi Man, Yubin Liu, Jie Zhao, Hegao Cai

**Affiliations:** 1 State Key Laboratory of Robot Technology and Systems, Harbin Institute of Technology, Harbin 150001, China; 21b908013@stu.hit.edu.cn (J.W.); 23b308002@stu.hit.edu.cn (Z.M.); jzhao@hit.edu.cn (J.Z.); hgcai@hope.hit.edu.cn (H.C.); 2 College of Intelligent Systems Science and Engineering, Harbin Engineering University, Harbin 150001, China; wangran407@hrbeu.edu.cn

**Keywords:** rehabilitation robot for balance disorders, gait phase identification, heuristic DNN, multi-sensor motion information fusion, variable admittance control strategy

## Abstract

This research study proposes a heuristic hybrid deep neural network (DNN) gait sub-phase recognition model based on multi-source heterogeneous motion data fusion which quantifies gait phases and is applied in balance disorder rehabilitation control, achieving a recognition accuracy exceeding 99%. Building upon this model, a motion control strategy for a novel rehabilitation training robot is designed and developed. For patients with some degree of independent movement, an active training strategy is introduced; it combines gait recognition with a variable admittance control strategy. This strategy provides assistance during the stance phase and moderate support during the swing phase, effectively enhancing the patient’s autonomous movement capabilities and increasing engagement in the rehabilitation process. The gait phase recognition system not only provides rehabilitation practitioners with a comprehensive tool for patient assessment but also serves as a theoretical foundation for collaborative control in rehabilitation robots. Through the innovative active–passive training control strategy and its application in the novel rehabilitation robot, this research study overcomes the limitations of traditional rehabilitation robots, which typically operate in a single functional mode, thereby expanding their functional boundaries and enabling more precise, personalized rehabilitation training programs tailored to the needs of patients in different stages of recovery.

## 1. Introduction

Gait sub-phases represent key stages in the walking cycle, such as the single-support phase and the swing phase. The classification of gait sub-phases is crucial to gait analysis, abnormal gait assessment, and rehabilitation plan design. For patients with balance disorders, the proportion and duration of each gait sub-phase reflect gait stability. A combined analysis of gait sub-phases and other gait features provides a comprehensive characterization of human movement, offering multidimensional data support for balance disorder rehabilitation [1,2,3,4]. Currently, gait recognition studies primarily rely on various sensor signals, integrated with technologies like computer vision, machine learning, and deep learning. For instance, Gao et al. employed multiple pressure sensors to collect foot–ground contact stress data, using an adaptive fuzzy neural network to segment gait phases [5]; Lee et al. gathered pressure data from the back, thighs, and feet, utilizing a Long Short-Term Memory (LSTM) recurrent neural network to map continuous percentage changes in gait phases [6]; Lu et al. predicted gait accurately by analyzing 64-channel electroencephalography (EEG) signals combined with feature extraction and K-means clustering algorithms [7]; Wang proposed a gait recognition method based on RGB and depth image fusion, enhancing the precision and robustness of gait identification [8]; Mogan et al. introduced an integrated gait recognition approach, combining multiple Convolutional Neural Networks (CNNs) and Transformer models to improve image-based gait recognition performance [9]; Hanif et al. developed a novel deep learning-based video gait classification framework, utilizing Particle Swarm Optimization (PSO) to adjust learning rates, achieving an accuracy of 94.14% on the CASIA-B public dataset [10]. A rehabilitation robot assists patients in restoring physical function by providing motion support. Rehabilitation robots are generally classified into two types: exoskeleton and end-effector robots. Exoskeleton robots are worn externally on the patient’s limbs, providing mechanical support and motion guidance, while end-effector robots help with rehabilitation exercises by using devices such as motion pedals, treadmills, or traction systems [11,12,13,14]. For example, Zuccon et al. developed a cable-suspended robot for early gait rehabilitation, utilizing multiple spring balance mechanisms to compensate for leg weight and facilitate supine walking practice [15]. Meng et al. designed a knee exoskeleton robot, constructing a knee joint torque model based on a Temporal Convolutional Network (TCN)–LSTM hybrid neural network and adapting joint impedance control parameters [16]. Penna et al. proposed a muscle-coordinated real-time intent decoding control method for a shoulder exoskeleton, inferring movement direction based on muscle coordination features to control the exoskeleton’s dynamics [17]. Xia et al. introduced a lower-limb exoskeleton control method based on fuzzy logic to minimize human–robot interaction forces while ensuring minimal trajectory tracking errors [18]. Hmaied et al. proposed a passive rehabilitation robot system for elbow and wrist joints, utilizing a constraint model predictive controller to ensure real-time, accurate trajectory tracking [19]. Khoshdel et al. developed a robust impedance control method for lower-limb rehabilitation robots based on electromyography (EMG), eliminating the need for the dynamic modeling of the patient–robot interaction and simplifying the control process [20]. Adeola et al. presented an adaptive impedance control method for upper-limb rehabilitation arms, estimating motion intent by adjusting torque and position sensors to control a single-degree-of-freedom (DOF) robotic arm for elbow flexion–extension [21]. In summary, gait analysis and recognition research primarily relies on bioelectrical data (e.g., EEG), physical motion data, and video/image data, employing traditional feature extraction and machine learning methods. With the advancement of deep learning, particularly the integration of CNNs and recurrent neural networks (RNNs), the accuracy of gait phase recognition has significantly improved, achieving recognition rates of 95–98%, or even higher, using standard gait recognition datasets such as CASIA-B and Kinect [22,23]. However, when applying gait recognition to robot control, issues such as real-time processing and computational complexity must be considered. Regarding rehabilitation robots and their control methods, exoskeleton robots offer more precise motion assistance and feedback but require more complex, customized designs and sensor control systems to accommodate individual patient differences and rehabilitation needs. In contrast, end-effector robots are safer and more adaptable, commonly used in the rehabilitation departments of medical institutions. However, existing end-effector robots often have limitations such as single-mode motion and a focus on localized joint rehabilitation, which may not meet the diverse needs of patients in different stages of recovery. Since patients exhibit varying motion characteristics in different stages of rehabilitation, enhancing the flexibility and adaptability of robot control remains a challenge. In previous work, we developed a novel 9-DOF rehabilitation training robot and explored a passive training strategy based on a washout algorithm (WA). This redundant structure and modular configuration offer larger motion space, improved flexibility, and better mechanical performance compared with traditional robots, providing a solid foundation for control algorithm development. Building upon this, we proposed an active training strategy based on gait phase recognition and admittance control. By incorporating gait phases into the robot control system, the robot can provide targeted support during different gait phases, assisting the patient during the stance phase and supporting them during the swing phase. This significantly enhances the functionality and adaptability of the rehabilitation robot, enabling personalized support for patients in various stages and with different rehabilitation needs. Experimental results demonstrate the high accuracy and effectiveness of the gait phase recognition system and active control strategy. This paper is structured as follows: 1—introduction to the rehabilitation robot, subsequently leading to the active and passive training strategy; 2—the gait phase recognition system, which is based on a heuristic hybrid deep learning model for high-precision gait phase recognition; 3—the passive training strategy, whereby natural gait data are extracted, and the WA is used to implement the passive training mode; 4—the active training strategy, whereby gait phase recognition and admittance control are used to provide assistance in the swing phase and support in the stance phase; 5—simulations and experiments.

## 2. Materials and Methods

### 2.1. A Novel Rehabilitation Robot System

**Figure 1:**
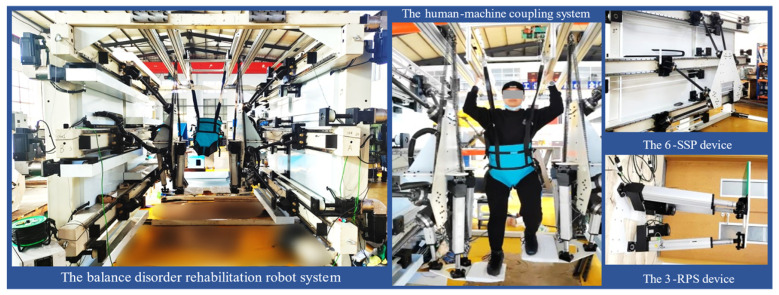
A novel rehabilitation robot system.

In previous research, we proposed a redundancy-driven, serial–parallel hybrid rehabilitation robot with 9 DOF for balance disorder rehabilitation. The robot features a modular design, comprising a 6-DOF parallel mechanism and a 3-DOF parallel mechanism. The 6-DOF mechanism consists of six motion chains, each configured as a spherical joint–spherical joint–prismatic joint arrangement, referred to as a 6-SSP device. The 3-DOF mechanism consists of three motion chains, each configured as a rotary joint–prismatic joint–spherical joint arrangement, referred to as a 3-RPS device. Two sets of 9-DOF rehabilitation robots are symmetrically arranged and combined with a suspension-based weight reduction system and virtual reality devices to form a comprehensive rehabilitation robot system [24,25]. The physical prototype of the robot is shown in Figure 1.

**Figure 2:**
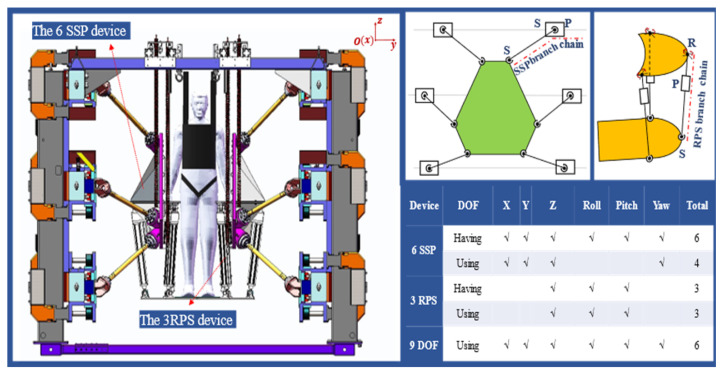
The 3D model of the robot and motion decomposition.

To fully leverage the motion advantages of the two modular devices and enhance the mechanical performance of the robotic system, motion decomposition principles for the 6-DOF and 3-DOF devices were established, as shown in Figure 2. During operation, the system allocates motion between the devices according to these principles, enabling independent kinematic calculations for each device, which then collaborate to complete the task. The end-effector of the dual 9-DOF rehabilitation robot is a motion platform, responsible for supporting and guiding the patient through rehabilitation exercises in a standing posture. This rehabilitation robot provides a larger range of motion, capable of simulating and assisting patients with various daily movement patterns. Its good dynamic performance also promotes full-body involvement in rehabilitation training, aiding in the recovery of motor perception and balance control abilities.

With the safety assurance provided by the suspension and weight reduction system, the rehabilitation robot can offer treatment for patients with various types and severities of balance disorders. In the early rehabilitation stages, when patients may be unable to stand or walk independently, the primary goal is to restore basic motor function. During this phase, the robot operates in a passive training mode (the patient is passive, and the robot is active), assisting patients with repetitive gait training. This mode facilitates the integration of the patient’s central nervous system, enhancing control and muscle strength in areas such as the pelvis and lower limbs. For patients in the functional recovery phase, who have some independent movement but lack the muscle strength to overcome external resistance and perform independent motions, a more refined interactive mode is employed to encourage active participation, known as the active training mode (the patient is active, and the robot is passive). The goal of this mode is to strengthen motor abilities, correct gait, and improve coordination. The robot’s combination of active and passive training modes provides flexible rehabilitation options, allowing for personalized treatment plans tailored to the patient’s physical condition and recovery needs.

This rehabilitation robot system is equipped with three safety protection mechanisms:

1. Software Limit Mechanism: In the upper computer software application, the motor position commands are limited based on the calculated working space of the robot, ensuring that the robot operates within an appropriate range at the software control level.
2. Hardware Limit Mechanism: Blocks are placed at appropriate positions at both ends of each guide rail slider to restrict the slider’s movement within a limited stroke.
3. Emergency Stop Device: During patient training, medical staff are required to supervise the process. In the event of an emergency, pressing the Emergency Stop Device will immediately activate the brakes on all motors, applying braking force to stop the motors. The robot will immediately stop working and remain stationary.

These safety mechanisms ensure the safe and reliable operation of the rehabilitation robot during training sessions.

### 2.2. Gait Phase Identification System

#### 2.2.1. Gait Phase Division and Multi-Sensor System Composition

**Figure 3:**
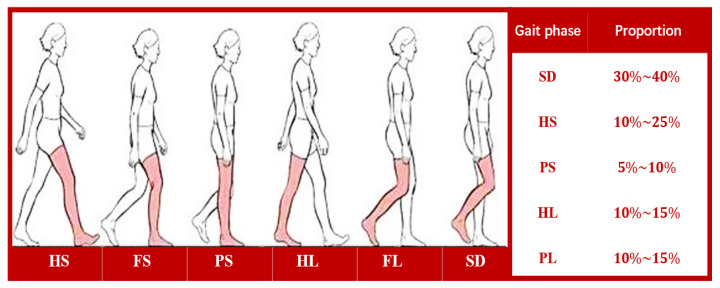
Subdivision of gait phases in gait cycle.

In individual locomotion, the gait cycle can be divided into several phases based on dynamic changes in the foot and leg. Traditional gait analysis typically categorizes the gait cycle into the stance phase and the swing phase, with the stance phase accounting for approximately 60–70%. During the alternation between the stance and swing phases, the single-support phase undergoes various gait stages, including sole dangling (SD), heel strike (HS), forefoot strike (FS), plantar support (PS), heel lift (HL), forefoot lift (FL), and the subsequent return to sole dangling. In this study, a gait cycle is divided into five sub-phases, SD, HS, PS, HL, and FL, as shown in Figure 3.

**Figure 4:**
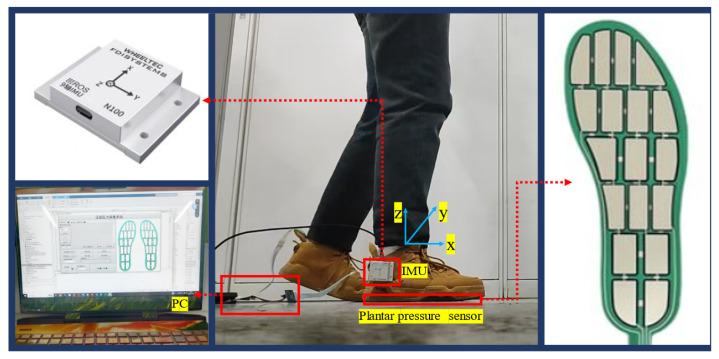
The composition of this multi-source heterogeneous sensing system.

Plantar pressure and ankle posture data serve as key signals for the gait phase recognition model, as illustrated in Figure 4. The plantar pressure sensors utilize an array-based, flexible thin-film pressure sensing technology, akin to insoles, positioned on the foot sole, with each sensor comprising 18 independent sensing units. The inertial measurement unit (IMU), a nine-axis posture sensor, captures changes in ankle orientation. By pairing plantar pressure sensors with IMUs, comprehensive motion data are collected. These data, acquired during natural gait by using wearable sensors, consist of 18 pressure channels and 1 posture channel, representing single-leg, multi-source heterogeneous information.

The technical specifications of the plantar pressure sensor and the posture sensor are presented in Table 1 and Table 2 below.

#### 2.2.2. Data Preprocessing

(1) Two-dimensional nonlinear interpolation based on Gaussian RBF

The flexible printed circuit–flexible matrix plantar pressure sensor consists of 18 pressure matrices for each foot. To enrich the data features and smooth the edges, a 2D nonlinear interpolation method based on the Gaussian Radial Basis Function (RBF) is applied to fit and expand the plantar pressure matrix. The RBF is a commonly used interpolation technique, particularly effective for nonlinear interpolation problems, as it can fit irregular data points and offers excellent smoothing properties [26,27,28]. In a 2D space, the commonly used RBF form is the Gaussian function, expressed mathematically as follows:

(1) $$f\left(r\right)=e^{-\frac{r^{2}}{2\sigma^{2}}}$$

where *r* is the distance between the input point and the interpolation point and σ is the smoothing parameter of the Gaussian function. The core of Gaussian RBF interpolation involves constructing a weighted sum function to represent the interpolation process. The interpolation approximation is given by Equation (2):

(2) $$\varphi \left(x,y\right)=\underset{i=1}{\sum^{N}}\;w_{i}\;\;\;\cdot \;\;\;f\left(\|\left(x,y\right)-\left(x_{i},y_{i}\right)\|\right)$$

where $w_{i}$ denotes the unknown weight coefficients, $\varphi \left(\|\left(x,y\right)-\left(x_{i},y_{i}\right)\|\right)$ is the weight value computed from the Gaussian function, and $\|\left(x,y\right)-\left(x_{i},y_{i}\right)\|$ is the Euclidean distance between the target point and the data point. To ensure that the interpolation function passes through all given data points, the weight coefficients are determined by solving the following linear system:

(3) A·w=b

where *A* is a matrix composed of the Gaussian function values, with elements $A_{ij}$ = $f\left(\|\left(x_{i},y_{i}\right)-\left(x_{j},y_{j}\right)\|\right)$, and *b* is the vector of known function values:

(4) $$b=\left\{f\left(x_{1},y_{1}\right),f\left(x_{2},y_{2}\right),\ldots ,f\left(x_{N},y_{N}\right)\right\}$$

**Figure 5:**
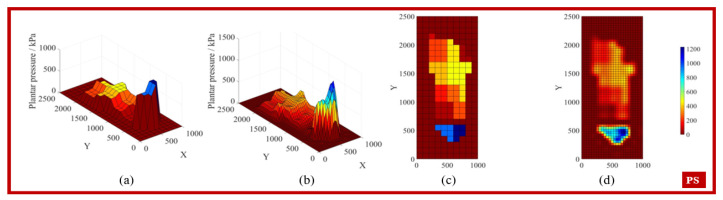
The plantar pressure in the PS phase.

**Figure 6:**
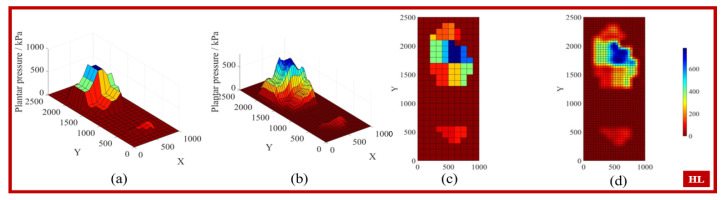
The plantar pressure in the HL phase.

**Figure 7:**
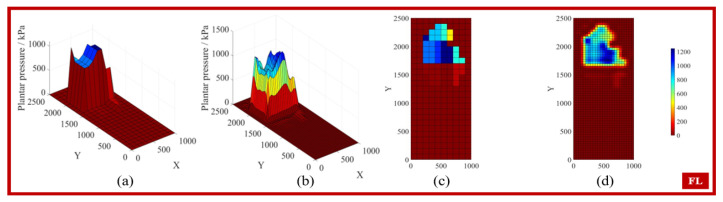
The plantar pressure in the FL phase.

**Figure 8:**
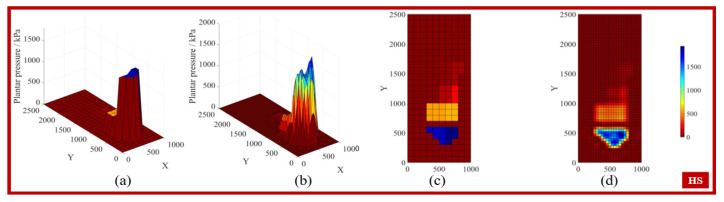
The plantar pressure in the HS phase.

By applying the interpolation conditions to search for the weight matrix $w_{i}$, the coefficients of the interpolation polynomial can be obtained, allowing the interpolation function to predict any point. Figure 5, Figure 6, Figure 7 and Figure 8 display the feature matrices of the plantar pressure before and after interpolation at specific time points, with the following visual representations: (a) 3D plot before interpolation, (b) 3D plot after interpolation, (c) top view before interpolation, and (d) top view after interpolation.

(2) Principal Component Analysis

Each plantar pressure sample consists of 26 × 63 features. To minimize data redundancy while preserving significant variability, Principal Component Analysis (PCA) is employed for feature extraction, enhancing the conciseness and effectiveness of the data for subsequent analysis [29,30]. The process begins with standardizing the raw data to eliminate dimensional discrepancies between features, as shown in Equation (5). Next, the covariance matrix is computed to examine the interrelationships among features, as illustrated in Equation (6) [31]. Subsequently, the eigenvalues and eigenvectors of the covariance matrix are derived, as shown in Equation (7), where eigenvectors define the new axes and the eigenvalues indicate variance along these directions. Finally, the eigenvectors corresponding to the largest eigenvalues are selected to construct a reduced-dimension space, and the original data are projected onto these principal components, as outlined in Equation (8).

(5) $$Z_{\mathrm{ij}}=\frac{X_{\mathrm{ij}}\cdot {\;\mu }_{j}}{{\;\sigma }_{j}}$$

(6) $$C=\frac{1}{m-1}\cdot Z^{T}\cdot Z.$$

(7) C·v=λ·v

(8) $$X^{\prime }=Z\cdot V_{k}$$

where the following apply:

$X_{\mathrm{ij}}$—the raw value of the i-th sample for the j-th feature.
$\mu_{j}$, $\sigma_{j}$—the mean and standard deviation of the j-th feature.
Z—the matrix of standardized data.
*m*—the number of samples.
*C*—the covariance matrix.
*v*—the eigenvector.
λ—the corresponding eigenvalue.

**Figure 9:**
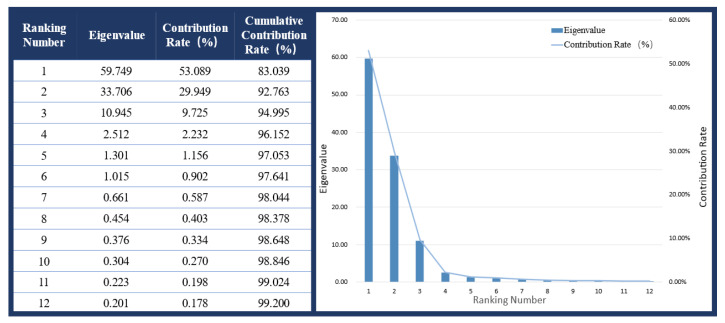
Principal Component Analysis results.

The reduced-dimension data $X^{\prime }$ are constructed from the original data *Z* and the eigenvectors of the selected *k* principal components, reducing the data from their original *n*-dimensional space to the *k*-dimensional space. Typically, *k* is chosen such that the cumulative variance contribution reaches a predetermined threshold (e.g., 95%) [32,33]. After applying PCA to the plantar pressure data, the relationships among the principal components, eigenvalues, and their contribution rates are shown in Figure 9 (sorted by eigenvalue, with the top 12 components displayed). The results indicate that six components have eigenvalues greater than 1, with values of 59.75, 33.71, 10.96, 2.51, 1.30, and 1.02, yielding a cumulative variance contribution of 97.64%. Additionally, the cumulative contribution of the first 11 components exceeds 99%. The eigenvalues of the remaining components rapidly decay to near zero, with minimal variance contribution. Thus, PCA successfully reduced the original 26 × 63 dimensional feature data to a lower-dimension space, eliminating redundant and irrelevant features while retaining the majority of the information. The reduced data, serving as input for the gait phase recognition model, significantly improve data processing efficiency.

(3) Gait phase identification system

**Figure 10:**
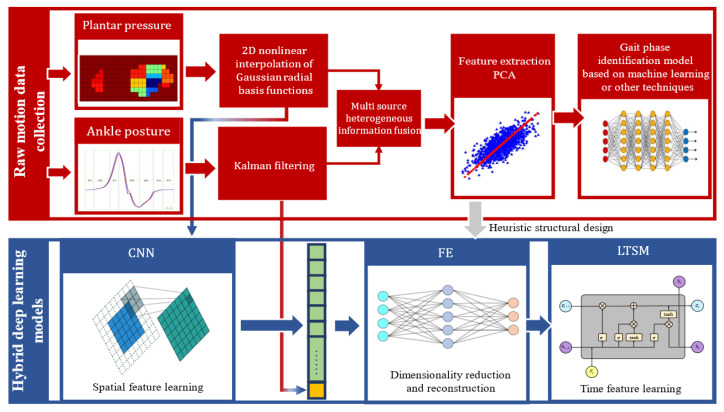
The heuristic structure of the hybrid deep learning model.

PCA has inherent limitations, primarily due to its reliance on the covariance matrix, which makes it sensitive to data scale and outliers, and its inability to effectively model nonlinear relationships. To address these shortcomings, this study proposes a deep learning-based gait phase recognition model that harnesses the nonlinear fitting power of neural networks while optimizing the network architecture based on PCA insights. The model features a hybrid neural network structure with three core components: a CNN, a feature extraction module (FE), and LSTM. The CNN component processes spatial features by progressively extracting spatial patterns from the plantar pressure data through convolutional layers while reducing spatial dimensions by using max pooling (MaxPool), enhancing feature abstraction, and reducing computational overhead. The feature extraction module compresses and reconstructs the CNN output to capture key information, ensuring efficient data representation and reducing network complexity. Based on PCA, the number of nodes is set to 10, with the top 10 principal components contributing over 98% of the variance, preserving essential information. Angular data are treated as supplementary features and concatenated with the CNN output to enhance the model’s multimodal learning capability. The fused data are then input into the LSTM component, which captures temporal dependencies in the time-series data. After extracting temporal features, the output is mapped to the target classification space via a fully connected (FC) layer, completing the gait classification task. Figure 10 illustrates the heuristic structure of this hybrid deep learning model.

### 2.3. Active Training Control Strategy

In passive training, the rehabilitation robot guides the patient through proper movement patterns to prevent incorrect postures, helping restore strength and coordination in relevant muscle groups and regain basic movement control. While the trajectory can be personalized to accommodate individual differences, this approach lacks active effort or motion adjustment, leading to reduced patient engagement. To improve rehabilitation outcomes and enhance robot functionality, we propose an active training control strategy based on gait phase recognition and admittance control. This strategy aims to increase patient autonomy, enhance involvement, and ultimately improve rehabilitation quality.

#### 2.3.1. Admittance Control

The robot defines its response to the interaction force between the patient and the robot through the concept of “admittance”. We integrate the admittance mechanism into an active control training strategy, using plantar pressure to sense the patient’s motion intentions and dynamically adjusting the robot’s trajectory based on this information. This feedback mechanism helps the patient maintain active participation during training, rather than passively following the robot’s guidance. Admittance control enables the robot to modulate between the guiding trajectory and the patient’s motion intention, adjusting the admittance parameters to change the robot’s compliance, thereby regulating the level of patient involvement [34,35,36,37,38]. Admittance control consists of an inner position control loop and an external admittance calculation loop. When the interaction force between the robot and the environment is $F_{HRI}$, the admittance model generates position correction ΔX. The reference trajectory of the robot’s end effector is $X_{r}$, which is adjusted to obtain the desired trajectory $X_{e}$. $X_{e}$ then enters the control inner loop, where a position controller ensures that the actual position *X* tracks $X_{e}$. The admittance controller is defined by Equation (9):

(9) $$-F_{e}=K_{d}\cdot \left(X-X_{r}\right)+B_{d}\cdot \left(\dot{X}-{\dot{X}}_{r}\right)+M_{d}\cdot \left(\ddot{X}-{\ddot{X}}_{r}\right)$$

**Figure 11:**
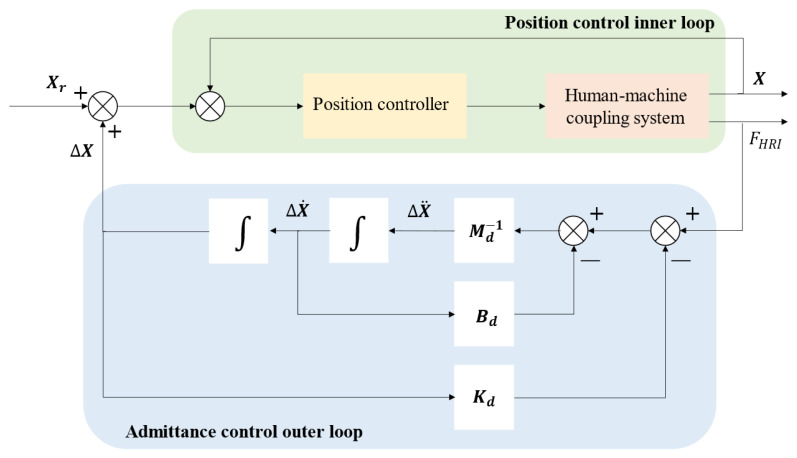
The control block diagram of admittance control.

$K_{d},B_{d}$, and $M_{d}$ represent the virtual stiffness, virtual damping, and virtual mass, respectively. By adjusting these parameters, the robot’s compliance can be varied, enabling diverse interactions with the environment. The admittance controller determines the output trajectory correction based on the human–robot interaction force and admittance parameters and works with the position controller to accurately track the desired trajectory. The control block diagram of admittance control is shown in Figure 11.

The dynamic model of the system can be expressed as

(10) $$M\left(q\right)\cdot \ddot{q}+C\left(q,\dot{q}\right)\cdot \dot{q}+G\left(q\right)=\tau$$

where the following apply:

*q*—the joint position vector of the robot.
$\dot{q}$—the joint velocity vector.
$M\left(q\right)$—the mass matrix.
$C\left(q,\dot{q}\right)$—the Coriolis and centrifugal force matrix.
$G\left(q\right)$—the gravitational torque.
τ—the control torque applied by the robot.

To prove the stability of the redundant robot’s active control, we apply the Lyapunov function *V*, defined as

(11) $$V=\frac{1}{2}{\left(q-q_{d}\right)}^{T}\cdot P\cdot \left(q-q_{d}\right)+\frac{1}{2}{\dot{q}}^{T}\cdot \dot{q}$$

where P—a symmetric positive definite matrix associated with system performance, defining the energy of the position error; $q_{d}$—the desired position; $\dot{q}$—a positive definite matrix. To verify system stability, we compute the time derivative of the Lyapunov function $\dot{V}$:

(12) $$\dot{V}={\left(q-q_{d}\right)}^{T}\cdot P\cdot \dot{q}+{\dot{q}}^{T}\cdot \ddot{q}$$

By substituting the admittance control law, we derive

(13) $$\dot{V}=-{\left(q-q_{d}\right)}^{T}\cdot K\cdot \left(q-q_{d}\right)-{\dot{q}}^{T}\cdot D\cdot q\;+{\dot{q}}^{T}\cdot M^{-1}\cdot F_{ext}$$

where $-{\left(q-q_{d}\right)}^{T}\cdot K\cdot \left(q-q_{d}\right)$ represents a negative definite term due to the stiffness matrix *K*, indicating that the system attempts to drive position errors to zero. $-{\dot{q}}^{T}\cdot D\cdot q$ represents a negative definite contribution from the damping matrix *D*, which helps to suppress oscillations and ensures that the velocity gradually approaches zero. The term ${\dot{q}}^{T}\cdot M^{-1}\cdot F_{ext}$ accounts for the effect of external forces on the system. If the external force $F_{ext}$ (or $F_{HRI}$) is bounded, we can infer that $\dot{V}$ will remain negative or semi-negative, suggesting that the system’s errors (both position and velocity) will progressively decrease. To ensure stability, we require $\dot{V}$ to be negative definite, which implies that

(14) $$\dot{V}\leq -a_{1}\cdot {\left|\left|q-q_{d}\right|\right|}^{2}-a_{2}\cdot {\left|\left|\dot{q}\right|\right|}^{2}$$

where $a_{1}$ and $a_{2}$ are positive constants. This condition indicates that the system’s energy will decrease over time, causing both position and velocity errors to converge to zero. Therefore, based on Lyapunov’s stability theory, if V is negative definite, the system will be stable, and the position and velocity errors will converge to zero. Provided that the admittance parameters are appropriately selected and $F_{HRI}$ remains within a controllable range, the system will maintain stability and converge to the desired state during interaction with the environment.

#### 2.3.2. Active Training Control Strategy Based on Gait Phase Switching

**Figure 12:**
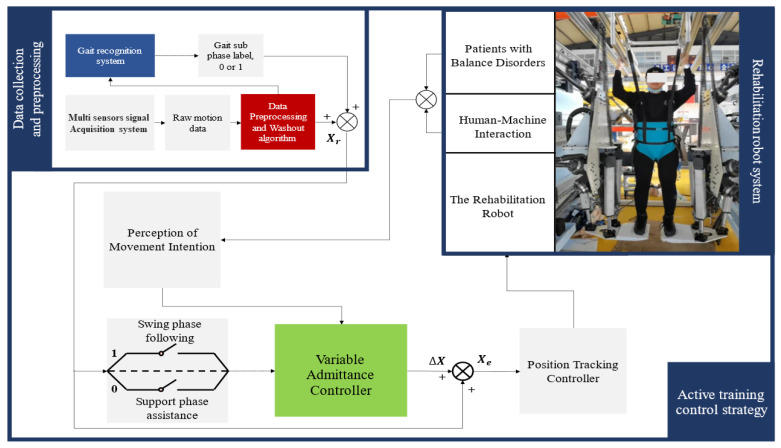
The active training control framework.

Walking is a cyclic process comprising alternating stance and swing phases. During the stance phase, one foot remains in contact with the ground, supporting body weight and maintaining stability, while the other foot lifts off, transitioning into the swing phase. Due to the distinct movement requirements of each phase, the robot must adjust its support and assistance accordingly. In the stance phase, the primary objective is to help the patient maintain stability and ensure vertical lower-limb alignment. In the swing phase, the robot facilitates rapid limb movement and coordination, which places higher demands on muscle strength and joint mobility. Here, the robot encourages active limb movement by reducing stiffness and increasing compliance. By continuously monitoring plantar pressure feedback, the robot can assess the patient’s movement state and dynamically adjust the level of assistance. Through the optimal adjustment of the admittance parameters, the robot offers personalized support based on the patient’s motor abilities, fatigue, and rehabilitation progress. To address this, we propose a variable admittance active training strategy, where assistance is provided during the stance phase and the robot adapts to the patient’s movements during the swing phase. This approach utilizes the previously mentioned heuristic hybrid deep neural network to accurately identify gait sub-phases, switching between high- and low-admittance controllers for the stance and swing phases. High admittance during the stance phase ensures stability and support, while low admittance during the swing phase facilitates limb movement, promotes gait training, and maintains continuity. Simultaneously, assistance is moderated to stimulate the patient’s active participation. Figure 12 illustrates the variable admittance active training control framework based on gait phase identification.

## 3. Experiments and Results

Based on theoretical research, we designed and implemented gait recognition experiments and built our own gait database. On this basis, experiments on passive and active training control strategies were performed.

### 3.1. Gait Phase Identification and Result Analysis

**Figure 13:**
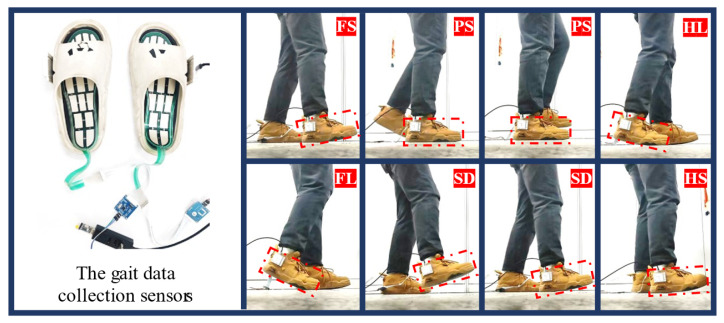
Gait motion data acquisition process.

**Figure 14:**
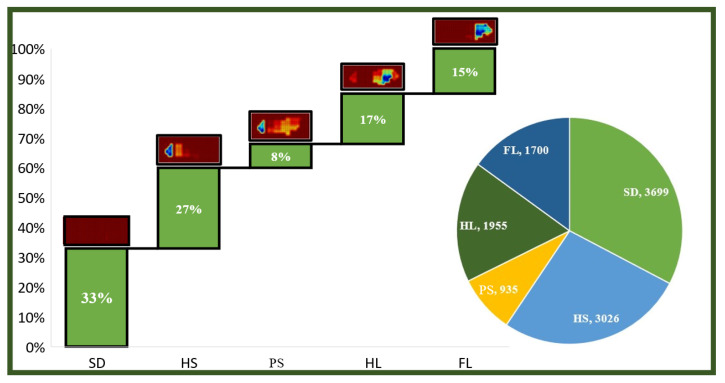
Raw gait data and their distribution.

Volunteers wore signal acquisition devices to perform walking experiments, during which a gait dataset was collected and constructed, as illustrated in Figure 13. The dataset includes 18 channels of single-foot plantar pressure grid data and 1 channel of posture data, comprising a total of 11,315 motion data points with a sampling period of 20 ms [39]. Based on the continuity of movement and posture characteristics, the data points were classified into five gait sub-phases. The distribution of gait sub-phases in the original dataset is shown in Figure 14.

**Figure 15:**
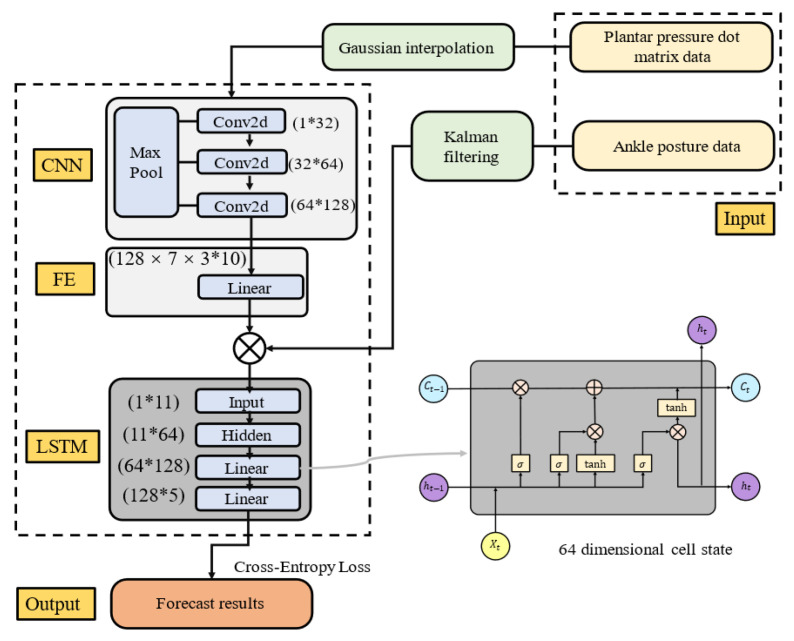
CNN–FE–LSTM.

Inspired by PCA, a network architecture highly aligned with the data features was designed. The CNN component extracts spatial features from the input data (plantar pressure images), which are then further reduced to a 10-dimensional feature space through the FE module. These features are subsequently fused with one-dimensional angular data for LSTM processing. The LSTM component uses a hidden layer with 64 units, followed by two fully connected layers to process the LSTM output, which is finally mapped to the target number of classes for the final classification result. To prevent overfitting, Dropout regularization is applied, and the Momentum and RMSProp optimization algorithms are used, demonstrating good convergence. The weights in both the CNN and LSTM are initialized by using a uniform distribution, with biases set to zero. ReLU activation is used in the fully connected layers, while the LSTM gates utilize the Sigmoid activation function, and the cell state update uses the Tanh function. The loss function is Cross-Entropy Loss, which calculates the logarithmic loss between the true and predicted labels and optimizes the model weights via backpropagation. The detailed architecture of the model is shown in Figure 15.

**Figure 16:**
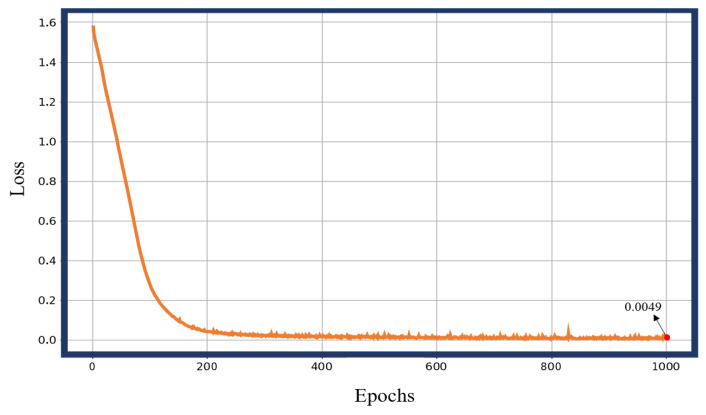
Loss iteration change.

**Figure 17:**
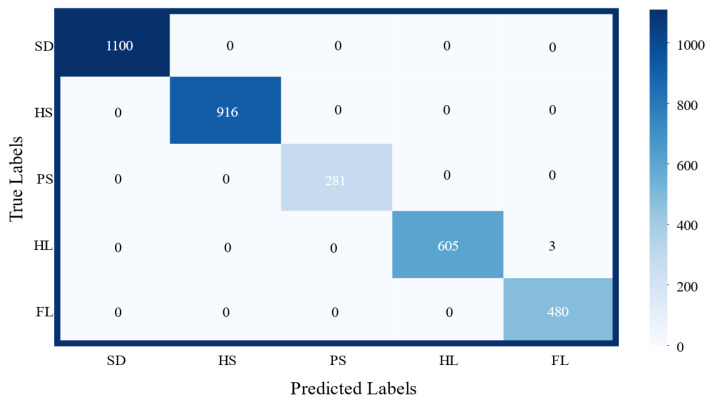
Classification confusion matrix.

The network model was implemented by using the PyTorch framework and trained on a self-built gait dataset. The dataset was split into training and validation sets in a 7:3 ratio, with a batch size of 64. The model was trained for 1000 epochs. Upon completion, the model achieved an accuracy of 99.82% on the training set and 99.91% on the test set. Figure 16 illustrates the loss curve across each epoch during training, while Figure 17 presents the classification confusion matrix on the test set at the end of training. The results indicate that misclassifications primarily occurred during the transitions between the HL and FL gait phases.

### 3.2. Active Training Control Strategy

An active training experiment based on gait phase recognition and adaptive admittance control was conducted for level walking. To ensure the versatility of the rehabilitation robot, the gait characteristics of its guiding trajectory (such as walking speed, stride length, etc.) and the training duration can be flexibly adjusted to accommodate different users. In the experiment, the robot moves along a predefined trajectory, with the admittance model calculating trajectory corrections. This model enables the robot to perceive the human motion intent and exhibit compliance, gradually reducing external assistance during rehabilitation and enhancing the patient’s awareness of active movement. Gait analysis revealed motion primarily in the X and Z directions, with minimal movement in the Y direction under ideal conditions. The human–robot interaction force is mainly reflected by the pressure exerted by the patient in the Z direction and the corresponding reaction force from the robot. Thanks to the washout algorithm, the robot’s continuous cyclic motion in the X direction is transformed into a periodic motion, reducing the interaction force. Since the patient’s primary effort is in the Z direction, an admittance controller is designed specifically for this direction. During rehabilitation, the robot provides sufficient stiffness in the stance phase to support the patient and maintain balance while exhibiting compliance in the swing phase to follow the patient’s movements and gradually reduce assistance, promoting the patient’s self-initiated efforts. The admittance model parameters can be adjusted according to individual patient capabilities and rehabilitation needs, enhancing the robot’s adaptability.

**Figure 18:**
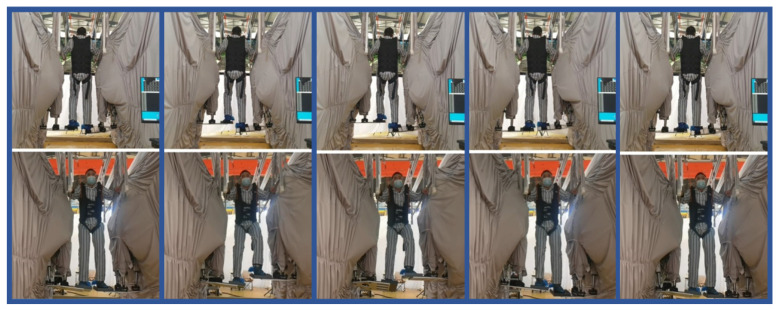
Active training experimental process.

Below, Figure 18 illustrates the active training experimental process. Volunteers were rigorously screened and evaluated to meet experimental requirements. The experiment was conducted under safety measures, including a suspension weight reduction device, ensuring safety. The robot underwent thorough calibration and system checks to ensure operational safety and reliability. During the experiment, the robot guided the volunteer along the planned trajectory, performing adjustments in real time via the admittance model to gradually reduce external assistance and enhance the patient’s autonomous movement capacity.

**Figure 19:**
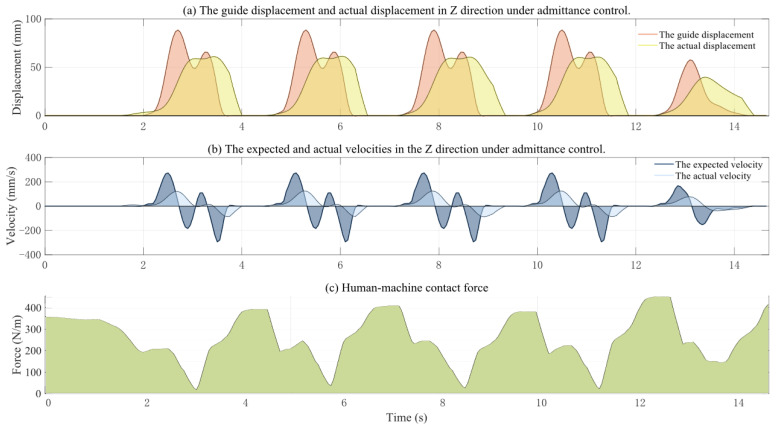
Changes in motion data during active training.

Figure 19 illustrates the variation in motion data during the active training process with gait phase transitions and adaptive admittance control, where the admittance parameters are set to M = 2, K = 40, and D = 18. Figure 19a shows the robot’s guided trajectory and the corrected trajectory, Figure 19b displays the robot’s guided velocity and the corrected velocity, and Figure 19c depicts the human–robot interaction force. From the figures, it can be observed that the effect of adaptive admittance on trajectory correction varies during the transition between the swing and stance phases. In the stance phase, the actual trajectory aligns with the corrected trajectory as the robot maintains rigid support to ensure stability. In the swing phase, due to the human–robot interaction force, the corrected trajectory deviates from the actual trajectory, demonstrating a degree of compliance. Due to the virtual damping effect, the system exhibits inherent time delay characteristics, which are unavoidable.

**Figure 20:**
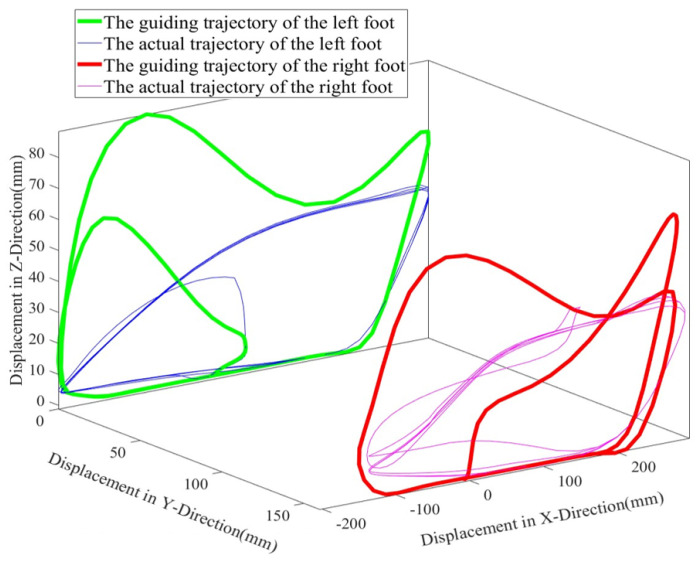
The predefined and actual motion trajectories.

Figure 20 shows the predefined and actual motion trajectories of the robot. The flexibility exhibited by the robot through admittance control allows for the appropriate correction of the motion trajectory.

## 4. Discussion and Conclusions

The proposed gait phase recognition model employs a hybrid CNN–FE–LSTM network optimized through PCA to refine the network structure. By focusing on principal components with high variance, the network effectively captures the relationships between components and target variables, enhancing both efficiency and data processing. Experimental results show that the hybrid DNN model achieves a recognition accuracy exceeding 99%. A novel rehabilitation robot, utilizing gait phase switching and a variable admittance active training control strategy, dynamically supports and assists the user by adjusting its movements to the patient’s gait. The robot adapts the guidance trajectory based on the patient’s active force levels during rehabilitation, engaging multiple body parts to promote recovery from balance disorders. Its efficacy was validated experimentally, offering a practical solution for rehabilitation robot control. Although the robot demonstrates significant advances, further improvements are required. Current research focuses on active training during flat-ground walking, with future work set to include additional exercise modes such as beach walking and surfing, along with corresponding active control strategies. Moreover, integrating multimodal sensors with virtual reality will enhance patients’ immersion and self-awareness, validating these approaches across diverse patient populations to improve the stability, practicality, and applicability of the control strategies. 

## Figures and Tables

**Table 1:** Performance parameters of plantar pressure sensor.

Performance Parameter	Value
Static Resistance	>1 MΩ
Working Voltage	3.3–5 V
Response Time	<20 ms
Hysteresis	<6%
Drift	<8%
Electromagnetic Interference	Nil

**Table 2:** Performance parameters of IMU sensors.

Parameter	Accelerometer	Gyroscope
Range	±16 g	±2000°/S
Linearity	<0.1% FS	<0.1% FS
Bandwidth	260 Hz	256 Hz
Orthogonality Error	±0.05°	±0.05°
Resolving Power	<0.5 mg	<0.02°/S

## Data Availability

The data can be obtained from the corresponding author upon reasonable request.
